# Supplementary material for: Multi‐dataset identification of innovative feature genes and molecular mechanisms in keratoconus
Source: J Cell Mol Med. 2024 Sep 19;28(18):e70079. doi: 10.1111/jcmm.70079 (PMC11412914; doi:10.1111/jcmm.70079)
Supplement: Supplementary file 2 — Material S2. [file JCMM-28-e70079-s002.zip › 7.GSEA analysis/5.RNF217-AS1/KEGG_GLYCOSAMINOGLYCAN_DEGRADATION.html]

Details for gene set KEGG\_GLYCOSAMINOGLYCAN\_DEGRADATION[GSEA]

|  || Dataset | RNF217-AS1.group.cls#h\_versus\_l |
| Phenotype | group.cls#h\_versus\_l |
| Upregulated in class | h |
| GeneSet | KEGG\_GLYCOSAMINOGLYCAN\_DEGRADATION |
| Enrichment Score (ES) | 0.2171161 |
| Normalized Enrichment Score (NES) | 0.67077535 |
| Nominal p-value | 0.85714287 |
| FDR q-value | 0.9732137 |
| FWER p-Value | 1.0 |
Table: GSEA Results Summary

  

Fig 1: Enrichment plot: KEGG\_GLYCOSAMINOGLYCAN\_DEGRADATION      
 Profile of the Running ES Score & Positions of GeneSet Members on the Rank Ordered List

  

| SYMBOL | TITLE | RANK IN GENE LIST | RANK METRIC SCORE | RUNNING ES | CORE ENRICHMENT || 1 | SPAM1 | na | 3444 | 0.273 | 0.0764 | Yes |
| 2 | HS3ST3A1 | na | 4106 | 0.249 | 0.2171 | Yes |
| 3 | HS3ST3B1 | na | 7635 | 0.137 | 0.2033 | No |
| 4 | ARSB | na | 11447 | 0.064 | 0.1343 | No |
| 5 | HYAL4 | na | 14344 | 0.026 | 0.0679 | No |
| 6 | HYAL3 | na | 14644 | 0.023 | 0.0741 | No |
| 7 | IDUA | na | 15130 | 0.018 | 0.0715 | No |
| 8 | GALNS | na | 16817 | 0.000 | 0.0232 | No |
| 9 | HEXA | na | 18191 | -0.013 | -0.0083 | No |
| 10 | HGSNAT | na | 19338 | -0.024 | -0.0262 | No |
| 11 | SGSH | na | 19558 | -0.026 | -0.0159 | No |
| 12 | GUSB | na | 19854 | -0.029 | -0.0061 | No |
| 13 | GNS | na | 21215 | -0.042 | -0.0182 | No |
| 14 | IDS | na | 21328 | -0.043 | 0.0065 | No |
| 15 | HPSE | na | 22583 | -0.057 | 0.0069 | No |
| 16 | HEXB | na | 22880 | -0.060 | 0.0372 | No |
| 17 | HYAL1 | na | 23159 | -0.064 | 0.0700 | No |
| 18 | HYAL2 | na | 23788 | -0.072 | 0.0982 | No |
| 19 | NAGLU | na | 24000 | -0.075 | 0.1405 | No |
| 20 | GLB1 | na | 26907 | -0.124 | 0.1362 | No |
| 21 | HPSE2 | na | 27594 | -0.139 | 0.2055 | No |
Table: GSEA details [plain text format]

  

Fig 2: KEGG\_GLYCOSAMINOGLYCAN\_DEGRADATION      
 Blue-Pink O' Gram in the Space of the Analyzed GeneSet

  

Fig 3: KEGG\_GLYCOSAMINOGLYCAN\_DEGRADATION: Random ES distribution      
 Gene set null distribution of ES for **KEGG\_GLYCOSAMINOGLYCAN\_DEGRADATION**
